# Postprandial levels of GLP-1, GIP and glucagon after 2 years of weight loss with a Paleolithic diet: a randomised controlled trial in healthy obese women

**DOI:** 10.1530/EJE-19-0082

**Published:** 2019-04-30

**Authors:** Julia Otten, Mats Ryberg, Caroline Mellberg, Tomas Andersson, Elin Chorell, Bernt Lindahl, Christel Larsson, Jens Juul Holst, Tommy Olsson

**Affiliations:** 1 Department of Public Health and Clinical Medicine, Umeå University, Umeå, Sweden; 2 Department of Food and Nutrition, and Sport Science, University of Gothenburg, Gothenburg, Sweden; 3 NNF Center for Basal Metabolic Research and Department of Biomedical Sciences, University of Copenhagen, Copenhagen, Denmark

## Abstract

**Objective:**

To investigate how weight loss by different diets impacts postprandial levels of glucagon-like peptide 1 (GLP-1), glucose-dependent insulinotropic polypeptide (GIP) and glucagon.

**Methods:**

In this single-centre, parallel group 2-year trial, 70 healthy postmenopausal obese women were randomised to the Paleolithic diet or a healthy control diet based on Nordic Nutrition Recommendations. Both diets were without calorie restriction. The primary outcome was the change in fat mass. Here, secondary analyses on GLP-1, GIP and glucagon measured during an OGTT are described.

**Results:**

In the Paleolithic diet group, mean weight loss compared to baseline was 11% at 6 months and 10% at 24 months. In the control diet group, mean weight loss was 6% after 6 and 24 months (*P* = 0.0001 and *P* = 0.049 for the comparison between groups at 6 and 24 months respectively). Compared to baseline, the mean incremental area under the curve (iAUC) for GLP-1 increased by 34 and 45% after 6 and 24 months in the Paleolithic diet group and increased by 59% after 24 months in the control diet group. The mean iAUC for GIP increased only in the Paleolithic diet group. The area under the curve (AUC) for glucagon increased during the first 6 months in both groups. The fasting glucagon increase correlated with the β-hydroxybutyrate increase.

**Conclusions:**

Weight loss caused an increase in postprandial GLP-1 levels and a further rise occurred during weight maintenance. Postprandial GIP levels increased only after the Paleolithic diet. Reduced postprandial glucagon suppression may be caused by a catabolic state.

## Introduction

Obesity is a major cause of cardiovascular disease and cancer, and its prevalence is increasing worldwide (1). Thus, it is critical to find effective treatments. An important issue is the regain of weight after different lifestyle interventions. This is at least partly due to resumption of previous lifestyle habits but also has physiological reasons. Importantly, diet composition may be important for weight loss maintenance with a high-protein and low-carbohydrate diet potentially being associated with improved body weight maintenance (2). Diet composition may also impact the secretion of hormones involved in the homeostatic regulation of body weight loss and weight maintenance.

A Paleolithic diet is based on fruit, vegetables, eggs, nuts, fish and lean meat and excludes salt, refined sugar, cereals and dairy products. By adhering to these recommendations, the Paleolithic diet contains compared to other diets less carbohydrate and more protein and unsaturated fat (3, 4, 5). A Paleolithic diet has been shown to improve glucose tolerance in individuals with ischaemic heart disease and HbA1c, triglycerides and blood pressure in patients with type 2 diabetes (3, 4, 6).

When nutrients pass through the intestine, enteroendocrine cells in the intestinal epithelium secrete the incretin hormones glucagon-like peptide-1 (GLP-1) and glucose-dependent insulinotropic polypeptide (GIP) into the circulation. Both incretins stimulate insulin secretion; GLP-1 also inhibits glucagon secretion and enhances satiety (7, 8, 9). Compared to lean individuals, overweight individuals show a reduced postprandial GLP-1 response (10, 11). On the other hand, GIP levels after food intake are either increased or similar in overweight compared to lean individuals (11, 12). Notably, it is not clear if alternations in incretin secretion are clinically important for weight loss and weight maintenance. Earlier investigations have thus reported both increasing and decreasing postprandial GLP-1 and GIP levels after diet-induced weight loss (13, 14, 15).

The pancreatic hormone glucagon is also involved in appetite regulation and is strongly regulated by plasma amino acids, suggesting that glucagon can be involved in appetite inhibition after protein-rich diets (16). When plasma glucose levels decrease, pancreatic alpha cells respond with increased glucagon secretion. In contrast, during glucose administration glucagon levels drop. This suppression of glucagon secretion is impaired in patients with type 2 diabetes and possibly also in overweight individuals (17).

Here, we report a 2-year study, in which we found excellent weight loss during the first 6 months and absence of weight regain during the rest of the study period. Our hypothesis was that postprandial incretin levels increase and postprandial glucagon levels remain unchanged during weight loss. By randomisation, we aimed to investigate if diet composition or weight loss *per se* affects postprandial incretin and glucagon levels.

## Subjects and methods

### Study design

For a 2-year period, overweight postmenopausal women were randomised to follow either a Paleolithic diet or a healthy control diet based on the Nordic Nutrition Recommendations.

### Subjects and randomisation

**Figure 1:**
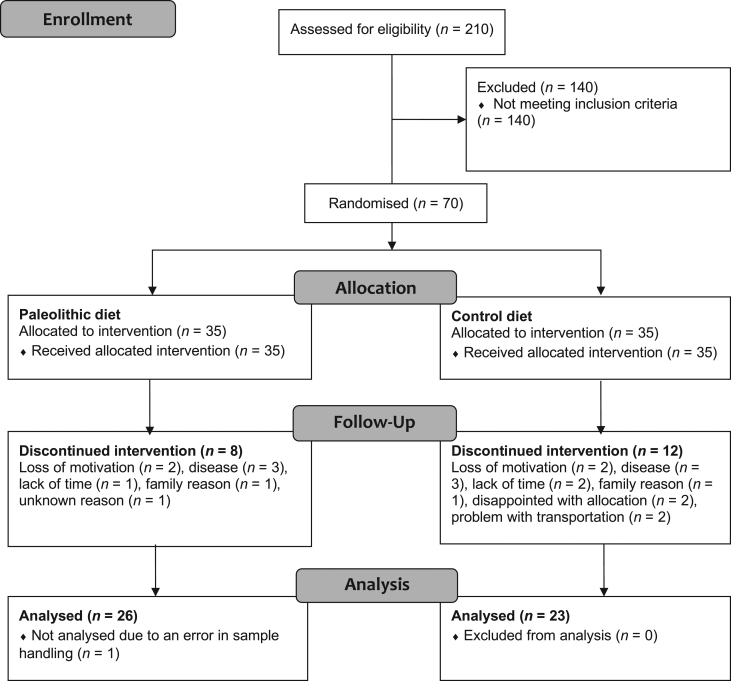
CONSORT flow diagram.

The subjects and the intervention have previously been described in detail (18). Briefly, healthy postmenopausal women (BMI of 27–41 kg/m^2^) were recruited using advertisements in local newspapers, and posters within the Umeå University Hospital area, between September 2007 and February 2008. The study was finished in 2010. Exclusion criteria were diabetes or fasting plasma glucose of ≥7 mmol/L; presence of severe disease; and treatment with statins, beta-blockers, hormone replacement or any medication for psychiatric disorders. A total of 70 women were randomised (Fig. 1). All study personnel, except the dieticians, were blind to the participants’ dietary allocations. The participants provided written informed consent, and the study was approved by the Regional Ethical Review Board at Umeå University.

### Diet intervention

After randomisation, study participants attended group sessions led by dieticians and cooking classes. Each individual participated in a total of 12 group sessions throughout the intervention period. Eight of the group sessions (including four cooking classes) took place during the first 6 months of the intervention. In both diets, energy intake was without calorie restriction. The Paleolithic diet was designed to increase protein intake to 30% of total energy (E%) and to provide 30 E% from carbohydrates and 40 E% from fat. It included fish, seafood, lean meat, eggs, nuts, vegetables, fruits, berries and vegetable oils, and excluded cereals, dairy products and added sugar and salt. The healthy control diet, a prudent diet based on the Nordic Nutrition Recommendations, was designed to decrease fat intake to 30 E% and to provided 55 E% from carbohydrates and 15 E% from protein (19). In this diet group, the women were advised to increase their intake of fruit, vegetables, whole grain and fish and to eat low-fat meat and dairy products.

### Measurements

In a fasting state, participants came for assessments at the Clinical Research Department, Umeå University Hospital. Fat mass was measured by dual-energy X-ray absorptiometry (GE Medical Systems). They were instructed to drink an oral glucose bolus of 75 g dissolved in water within 5 min. Blood samples were drawn prior to glucose ingestion, and every 30 min for a total of 2 h. Plasma glucose and insulin were immediately analysed at the Department for Clinical Chemistry, Umeå University Hospital. For other analyses, plasma samples were stored at −80°C and analysed in batch in January 2014.

Glucagon was measured using a radioimmunological assay directed against the COOH terminus, as previously described (20, 21, 22). Total plasma GLP-1 concentration (intact GLP-1 plus the metabolite GLP-1 9-36 amide) was also assayed via a previously described radioimmunological method (23, 24). The analytical detection limit for both glucagon and GLP-1 was 1 pmol/L. Total plasma GIP concentration (the sum of intact GIP plus the metabolite GIP 3-42) was measured by radioimmunoassay, as previously described (24, 25). The AUC was calculated using the trapezoidal rule, and both total AUC and incremental (baseline subtracted) AUC were determined. Glucagon decreases during the OGTT and only the total AUC was used.

The plasma concentration of β-hydroxybutyrate was measured using gas chromatography coupled to mass spectrometry (GC-MS). Prior to GC-MS analysis, plasma samples were extracted with methanol/water (90/10, v/v) and spiked with isotope-labelled internal standards, followed by a two-step derivatisation procedure (26). The derivatised samples (1 μL) were analysed on an Agilent 6890 gas chromatograph, equipped with a 10 m × 0.18 mm i.d. fused silica capillary column with a chemically bonded 0.18-µm DB 5-MS stationary phase (J&W Scientific, Folsom, CA, USA). The injector temperature was 270°C. The column temperature was held at 70°C for 2 min, increased by 40°C/min to 320°C and held there for 1 min. The column effluent was introduced into the ion source of a Pegasus III time-of-flight mass spectrometer (GC-TOF/MS; Leco, St. Joseph, MI, USA). The transfer line temperature was 250°C, and the ion source temperature was 200°C. Ions were generated by a 70-eV electron beam at a 2.0-mA ionisation current. Spectra were recorded in the mass range 50–800 m/z, at a rate of 30 spectra/s. The unique mass-to-charge (m/z) ratio of 191 was used to quantify β-hydroxybutyrate. This method gives no exact concentration but relative concentrations that enable comparison between groups/timepoints.

The plasma concentration of non-esterified fatty acids was analysed using the NEFA-HR_2_ kit (Wako Chemicals).

Energy and macronutrient intake were assessed by 4-day self-reported food diaries. For validation of protein intake study participants collected three 24-h urine samples for analysis of nitrogen excretion at baseline, 6 months and 24 months as reported earlier (18). Analytical precision was ±10%.

### Power analysis, randomisation and statistical analysis

We performed a secondary analysis of the 2-year diet intervention (18). The primary outcome of the study was the change in fat mass over a period of 2 years. Power calculation based on this objective indicated that 35 subjects were needed in each intervention arm to achieve a significant outcome (*P* < 0.05) with 80% power. In a previous study of 20 obese individuals, weight loss altered postprandial GLP-1 and GIP levels (14), indicating that we should have enough power for the secondary analysis described in this paper, despite the relatively high loss-to-follow-up rate. Randomisation of this single-centre, parallel group trial was performed by a statistician blinded to the study, using a block size of four and a 1:1 allocation ratio. Baseline values and treatment effects (change from baseline to 6 months and from baseline to 24 months) were compared between groups using the Mann–Whitney *U* test. Within-group changes over time from baseline to 6 months and from baseline to 24 months were analysed using the Wilcoxon signed-rank test. Correlation analyses were performed by calculating Spearman’s rho (r_S_). A two-sided *P* value of <0.05 was considered statistically significant. Statistical analyses were performed using SPSS 24.0 for Mac (IBM Corp). Data are presented as mean ± s.e.m. unless otherwise stated.

## Results

### Dietary adherence

A previous publication describes the dietary adherence, and effects of each diet on parameters of the metabolic syndrome (18). During the intervention both diet groups decreased their energy intake (Table 1). According to food diaries, the Paleolithic diet group decreased carbohydrate intake more than the control diet group (*P* = 0.0003 and *P* = 0.002 for the comparison between groups at 6 and 24 months respectively, Table 1). At 6 months, fat intake in the control diet group decreased more than that in the Paleolithic diet group (*P* < 0.0001 for the comparison between groups, Table 1).

**Table 1:** Dietary intake according to food records at baseline and during 24 months of following a Paleolithic diet (*n* = 26) or a healthy control diet based on Nordic Nutrition Recommendations (*n* = 22). Data are presented as mean ± S.E.M.

	Paleolithic diet			Control diet		
	Baseline	6 months	24 months	Baseline	6 months	24 months
Energy intake						
kJ/day	8221 ± 251	6790 ± 255^###^	6308 ± 377^###^	8699 ± 356	6635 ± 293^###^	7183 ± 268^###^
kcal/day	1964 ± 60	1622 ± 61^###^	1507 ± 90^###^	2078 ± 85	1585 ± 70^###^	1716 ± 64^###^
*n*	26	26	23	22	19	19
Carbohydrate intake						
g/day	220 ± 7	119 ± 6***^,###^	116 ± 10**^,###^	231 ± 11	175 ± 7^###^	185 ± 12^###^
E%	45.6 ± 0.6	30.0 ± 1.3***^,###^	31.0 ± 1.7***^,###^	45.0 ± 0.9	45.1 ± 1.3	43.8 ± 2.3
*n*	26	26	23	22	19	19
Fat intake						
g/day	74 ± 3	79 ± 5***	71 ± 5	81 ± 4	55 ± 4^###^	66 ± 4^#^
E%	33.3 ± 0.6	42.6 ± 1.3***^,###^	41.6 ± 1.6**^,###^	34.4 ± 0.8	30.1 ± 1.2^#^	34.2 ± 1.9
*n*	26	26	23	22	19	19
Protein intake						
g/day	83 ± 3	93 ± 3***^,##^	85 ± 4*	85 ± 3	75 ± 3^#^	72 ± 2^#^
E%	17.2 ± 0.3	23.6 ± 0.6***^,###^	23.3 ± 0.8***^,###^	16.9 ± 0.6	19.6 ± 0.8^##^	17.3 ± 0.5
*n*	26	26	23	22	19	19
Protein intake^†^						
g/day	100 ± 5	98 ± 4	88 ± 6	102 ± 4	94 ± 5^#^	88 ± 4
E%	18.2 ± 1.0	21.4 ± 1.0^##^	21.9 ± 2.1^#^	17.9 ± 1.1	21.7 ± 2.0^##^	17.9 ± 1.0
*n*	25	23	18	20	17	16

*P < 0.05, **P < 0.01, ***P < 0.001 for the difference between diet groups. ^#^*P* < 0.05, ^##^*P* < 0.01, ^###^*P* < 0.001 for the within-group change over time vs baseline. ^†^According to nitrogen excretion.

According to measurements of nitrogen excretion, both diet groups increased protein intake (E%) during the first 6 months of intervention, but after 24 months only the Paleolithic diet group had increased protein intake (E%) compared to baseline (Table 1). Protein intake (E%) reported by the participants in food diaries showed comparable results (Table 1). Reported protein intake (E%) and measured protein intake by nitrogen excretion (E%) were highly correlated (*r*_S_ = 0.54, *P* < 0.0001).

### Body weight and fat mass

Baseline characteristics did not significantly differ between groups (Table 2). Individuals following the Paleolithic diet lost significantly more weight than those in the control diet group (*P* = 0.0001 and *P* = 0.048 for the comparison between groups at 6 and 24 months respectively, Table 2). In the Paleolithic diet group, the mean weight loss compared to baseline was 11% after 6 months and 10% after 24 months. In the control diet group, the mean weight loss compared to baseline was 6% after both 6 and 24 months. In the Paleolithic diet group, fat mass compared to baseline decreased by 19% after 6 months and by 14% after 24 months (Table 2). This decrease of fat mass was more pronounced compared to that observed in the control diet group (decrease of 10% after 6 months and 9% after 24 months).

**Table 2:** Patient characteristic, fasting plasma β-hydroxybutyrate (BHB) and non-esterified fatty acids (NEFA) at baseline and during 24 months of following a Paleolithic diet (*n* = 26) or a healthy control diet based on Nordic Nutrition Recommendations (*n* = 23). Data are presented as mean ± s.e.m. Values within parentheses indicate number (*n*).

	Paleolithic diet			Control diet		
	Baseline	6 months	24 months	Baseline	6 months	24 months
Age (years)	61 ± 1			61 ± 1		
Weight, kg	85.5 ± 2.1 (26)	76.2 ± 2.1(25)***^,###^	77.3 ± 2.3 (26)*^,###^	84.9 ± 1.6 (23)	80.1 ± 1.8 (23)^###^	80.1 ± 2.0 (22)^###^
BMI, kg/m^2^	32.5 ± 0.8 (26)	28.9 ± 0.8 (25)***^,###^	29.3 ± 0.8 (26)*^,###^	32.1 ± 0.5 (23)	30.3 ± 0.6 (23)^###^	30.4 ± 0.7 (22)^###^
Fat mass, kg	39.2 ± 1.5 (26)	31.9 ± 1.5 (26)***^,###^	33.8 ± 1.6 (23)*^,###^	39.2 ± 1.1 (23)	35.3 ± 1.4 (23)^###^	35.7 ± 1.5 (22)^###^
Fasting plasma BHB^†^	3.8 ± 0.7 (25)	5.8 ± 1.3 (25)*^,#^	5.3 ± 0.9 (21)	3.0 ± 0.4 (22)	3.4 ± 0.8 (21)	4.1 ± 1.1 (22)
Fasting NEFA (mmol/L)	0.48 ± 0.04 (26)	0.42 ± 0.03 (26)	0.40 ± 0.04 (26)	0.50 ± 0.03 (23)	0.45 ± 0.03 (23)	0.45 ± 0.04 (22)

*P < 0.05, ***P < 0.001 for the difference between diet groups. ^#^*P* < 0.05, ^###^*P* < 0.001 for the within-group change over time vs baseline. ^†^Values represent relative concentration.

### Glucose and insulin

**Figure 2:**
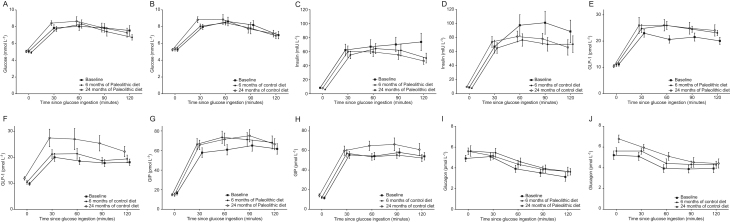
Measurements of glucose (A and B), insulin (C and D), GLP-1 (E and F), GIP (G and H) and glucagon (I and J) during an oral glucose tolerance test after 6 and 24 months following a Paleolithic diet (A, C, E, G and I) or a healthy control diet based on the Nordic Nutrition Recommendations (B, D, F, H and J). Data are presented as mean ± s.e.m.

Fasting glucose, fasting insulin and surrogate measures of insulin sensitivity are published in a separate paper (27). Neither group showed changes in fasting glucose or glucose levels after oral glucose bolus ingestion during the intervention (Fig. 2A, B and Table 3). Fasting insulin decreased between baseline and 6 months, only in the Paleolithic diet group (Table 3). The incremental area under the curve (iAUC) for insulin showed a tendency of declining between baseline and 24 months in both intervention groups (*P* = 0.10 for the Paleolithic diet group and *P* = 0.37 for the control diet group; Fig. 2C, D and Table 3).

**Table 3:** Hormone profiles during an oral glucose tolerance test at baseline and over 24 months of following a Paleolithic diet (*n* = 26) or a healthy control diet based on Nordic Nutrition Recommendations (*n* = 23). Data are presented as mean ± s.e.m. Values within parentheses indicate number (*n*).

	Paleolithic diet			Control diet		
	Baseline	6 months	24 months	Baseline	6 months	24 months
Glucose						
Total AUC (120 min × mmol/L)	902 ± 47 (25)	879 ± 43 (25)	935 ± 46 (26)	927 ± 43(22)	913 ± 46 (22)	949 ± 48 (21)
Incremental AUC (120 min × mmol/L)	299 ± 36 (25)	296 ± 34 (25)	329 ± 32 (26)	309 ± 36 (22)	290 ± 35 (22)	322 ± 35 (21)
Fasting concentration (mmol/L)	5.1 ± 0.2 (25)	4.9 ± 0.1 (26)	5.1 ± 0.2 (26)	5.3 ± 0.2 (23)	5.3 ± 0.2 (23)	5.2 ± 0.1 (22)
Insulin						
Total AUC (120 min × mU/L )	7233 ± 952 (26)	6310 ± 644 (26)	6080 ± 600 (26)	9452 ± 1163 (22)	7545 ± 802 (22)	8151 ± 812 (21)
Incremental AUC (120 min × mU/L)	6219 ± 887 (26)	5582 ± 613 (26)	5134 ± 537 (26)	8371 ± 1080 (22)	6598 ± 769 (22)	6981 ± 741 (21)
Fasting concentration (mU/L)	8.5 ± 0.8 (26)	6.1 ± 0.4 (26)^###^	7.9 ± 0.7 (26)	8.8 ± 0.9 (23)	7.9 ± 0.7 (23)	9.8 ± 0.9 (22)
GLP-1						
Total AUC (120 min × pmol/L)	2424 ± 141 (24)	2769 ± 122 (25)^##^	2826 ± 244 (20)^#^	2113 ± 132 (18)	2287 ± 169 (21)	2907 ± 325 (18)^##^
Incremental AUC (120 min × pmol/L)	1084 ± 99 (24)	1449 ± 111 (25)^##^	1572 ± 233 (20)^#^	933 ± 106 (18)	1036 ± 134 (21)	1488 ± 266 (18)^#^
Fasting concentration (pmol/L)	11.2 ± 0.7 (24)	10.9 ± 0.6 (26)	10.8 ± 0.7 (22)*	10.1 ± 0.6 (22)	10.4 ± 0.7 (22)	11.9 ± 0.6 (20)^##^
GIP						
Total AUC (120 min × pmol/L)	6683 ± 559 (24)	7580 ± 664 (26)^#^	7593 ± 593 (18)^#^	6021 ± 371 (20)	5953 ± 355 (21)	6858 ± 567 (18)
Incremental AUC (120 min × pmol/L)	4708 ± 426 (24)	5804 ± 622 (26)^#^	5799 ± 540 (18)^##^	4629 ± 349 (20)	4503 ± 334 (21)	5151 ± 534 (18)
Fasting concentration (pmol/L)	16.5 ± 1.9 (24)	14.8 ± 1.2 (26)	16.9 ± 1.9 (21)	12.3 ± 1.2 (22)	12.1 ± 1.3 (21)	14.4 ± 1.5 (20)
Glucagon						
Total AUC (120 min × pmol/L)	496 ± 36 (23)	558 ± 42 (24)^#^	539 ± 34 (20)	526 ± 42 (18)	580 ± 37 (21)^#^	636 ± 37 (19)
Fasting concentration (pmol/L)	5.0 ± 0.3 (24)	5.5 ± 0.5 (25)	5.7 ± 0.4 (22)	5.5 ± 0.4 (22)	5.6 ± 0.3 (22)	6.8 ± 0.4 (21)^#^
Fasting glucagon/insulin ratio (pmol/mU)	0.65 ± 0.06 (23)	1.03 ± 0.10 (24)^##^	0.93 ± 0.11 (20)	0.70 ± 0.09 (17)	0.87 ± 0.11 (21)^##^	0.88 ± 0.09 (18)^##^

*P < 0.05 for the difference between diet groups. ^#^*P* < 0.05, ^##^*P* < 0.01, ^###^*P* < 0.001 for the within-group change over time vs baseline.

### Glucagon-like peptide-1

Fasting GLP-1 levels increased only in the control diet group (Table 3).

In the Paleolithic diet group, the mean iAUC of GLP-1 increased by 34% after 6 months and by 45% after 24 months compared to baseline (Fig. 2E and Table 3). In the control diet group, the mean iAUC of GLP-1 did not increase after 6 months but increased by 59% after 24 months compared to baseline (Fig. 2F). The increase of the mean iAUC for GLP-1 from 6 to 24 months was significant in the control diet group (*P* = 0.04) but not in the Paleolithic diet group (*P* = 0.75), but did not significantly differ between diet groups.

### Association between glucagon-like peptide-1, body weight and food intake

During the first 6 months of control diet, the increase of fasting GLP-1 was associated with decreased carbohydrate intake (*r*_S_ = −0.62, *P* = 0.02) and increased fat intake (*r*_S_ = 0.54, *P* = 0.048). Body weight or protein intake at baseline or during the intervention was not associated with fasting GLP-1 levels.

**Figure 3:**
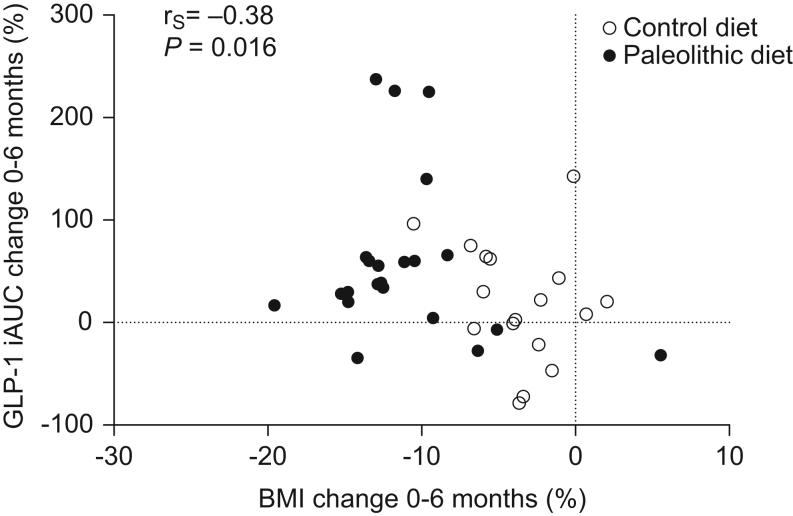
Association between changes observed in BMI and iAUC GLP-1 during 6 months of Paleolithic diet (*n* = 22) or a healthy control diet based on the Nordic Nutrition Recommendations (*n* = 17).

Postprandial GLP-1 levels were inversely correlated with BMI at baseline (*r*_S_ = −0.42, *P* = 0.005). Assessment of the whole study population revealed that the increase in postprandial GLP-1 between baseline and 6 months was associated with weight loss (Fig. 3). This correlation was not significant when each intervention group was analysed separately. Protein intake (measured by nitrogen excretion), fat and carbohydrate intake (reported by the participants) were not associated with postprandial GLP-1 levels at baseline or during the intervention.

### Glucose-dependent insulinotropic polypeptide

Fasting GIP levels did not change during the diet intervention.

The mean iAUC for GIP increased significantly by 23% after 6 and 24 months compared to baseline in the Paleolithic diet group (Fig. 2G and Table 3). Among controls, the mean iAUC for GIP increased slightly (11%) after 24 months (Fig. 2H). The mean iAUC for GIP did not significantly differ between diet groups.

### Association between glucose-dependent insulinotropic polypeptide, body weight and food intake

In the study population as a whole, the increase of iAUC GIP levels after 6 months was not associated with weight changes but with decreased carbohydrate intake (*r*_S_ = −0.46, *P* = 0.003). This correlation was non-significant when each intervention group was analysed separately. During the first 6 months of Paleolithic diet, increased protein intake (E% according to nitrogen excretion) tended to be correlated with increased postprandial GIP levels (*r*_S_ = 0.41, *P* = 0.06). Fat intake was not associated with postprandial GIP levels.

Changes of postprandial GIP levels during the intervention correlated with changes of postprandial GLP-1 levels (*r*_S_ = 0.47, *P* = 0.003).

### Glucagon

Fasting glucagon increased slightly at 6 months in both diet groups (*P* = 0.055 for the Paleolithic diet group and *P* = 0.052 for the control diet group) with a significant increase of 24% between baseline and 24 months in the control diet group only. The fasting glucagon/insulin ratio increased after 6 months compared to baseline in both groups, which was sustained after 24 months in the control diet group (Table 3).

During the first 6 months, the mean total AUC for glucagon increased by 13% in the Paleolithic diet group and by 10% in the control diet group (Fig. 2I, J and Table 3). There was a tendency for postprandial glucagon to further increase after 24 months in the control diet group (*P* = 0.12).

### Association between glucagon and body weight, food intake, β-hydroxybutyrate and non-esterified fatty acids

In the Paleolithic diet group, increasing fasting glucagon levels during 24 months of intervention were correlated with decreasing carbohydrate intake (*r*_S_ = −0.50, *P* = 0.04) and increasing fat intake (*p*_S_ = 0.55, *P* = 0.02). This association was not found in the control group. Fasting glucagon levels were not associated with body weight or protein intake (according to nitrogen excretion) at baseline or during the intervention.

The ketone body β-hydroxybutyrate increased at 6 months in the Paleolithic diet group, with a significant difference versus the control diet group (Table 2). This increase in ketone bodies was associated with increased fasting glucagon (*r*_S_ = 0.36, *P* = 0.03) and the increased glucagon/insulin ratio (*r*_S_ = 0.46, *P* = 0.006) within the whole study population. Fasting non-esterified fatty acids did not change significantly during the intervention, but the increase in ketone bodies was associated positively with changes in fasting non-esterified fatty acids during 6 months (*r*_S_ = 0.32, *P* = 0.03) and 24 months of intervention (*r*_S_ = 0.38, *P* = 0.01).

Postprandial glucagon levels were not associated with body weight, protein, fat or carbohydrate intake at baseline or during the intervention.

## Discussion

We have found a potential explanation for the excellent diet-induced long-term weight reduction in the present cohort of obese postmenopausal women (18). The intervention caused an increase in postprandial GLP-1 levels that was associated with weight loss but not with macronutrient composition of the diet. Interestingly, postprandial GLP-1 levels further increased during up to two years of weight maintenance. Moreover, the Paleolithic diet intervention led to an increased GIP response to glucose that was not associated to changes in body weight but to changes in macronutrient composition of the diet. We also detected a concomitant slight increase in fasting glucagon levels, which was reflected in less suppressed postprandial AUCs and was not associated with weight loss.

Weight loss during the first 6 months of our study caused an increase in postprandial GLP-1 levels, which was significant only in the Paleolithic diet group. Interestingly, postprandial GLP-1 levels increased even further between 6 and 24 months when body weight remained stable (now significant also in the control group). Two earlier studies in healthy obese individuals have shown comparable results: increasing postprandial GLP-1 levels after weight loss and a further increase after weight maintenance (13, 14). However, other weight loss studies in healthy obese individuals showed unchanged or decreased postprandial GLP-1 levels (28, 29, 30).

There are several possible explanations for these divergent results. The method used for GLP-1 measurement may be a major contributing factor. Two studies measured only active GLP-1 (28, 29). Active GLP-1 is rapidly degraded to the inactive form (t_1/2_ ≈ 1.5 min), such that only 10% of secreted GLP-1 ever reaches the circulation in its active form (31). Thus, it is very difficult to detect changes in GLP-1 secretion using this method. Here we measured total GLP-1, including both the active form and the primary GLP-1 metabolite, which provides a better estimate of GLP-1 secretion (23). It is also notable that all three weight loss studies that found decreasing or unchanged postprandial GLP-1 were short-term studies of very low-calorie diets (28, 29, 30). An acute negative energy balance on the examination day or in the days before measurement may cause increased plasma levels of non-esterified fatty acids, which could decrease GLP-1 secretion (32). In this scenario, a shorter study period might induce smaller changes that would likely become apparent over time given that the AUC for GLP-1 increases further during a period of weight loss maintenance. Although it is presently unclear how postprandial GLP-1 levels change during periods of negative energy balance and concomitant rapid weight loss, the available evidence suggests that postprandial GLP-1 increases after weight loss followed by a longer period of neutral energy balance and weight maintenance. It is possible that GLP-1 may be key to preventing weight regain via efficient diet interventions, as the postprandial increases after weight loss may contribute to increased satiety.

The Paleolithic diet group also exhibited increased postprandial GIP levels, but this increase was not associated with weight loss. Three previous studies also report increased postprandial GIP levels after short-term weight loss with a very low-calorie diet (14, 28, 30). However, increased, decreased and unchanged postprandial GIP levels are reported after weight maintenance (13, 14, 28). Notably, we did not find any significant changes of postprandial GIP levels in our control diet group. The more pronounced weight loss in the Paleolithic diet group compared to the control diet group may thus contribute to this result. However, factors other than weight loss may be responsible for the increase in postprandial GIP levels in the Paleolithic diet group. Studies in rodents demonstrate that dietary fat consumption chronically stimulates GIP production and secretion and even induces hyperplasia of GIP-producing K cells (33). In our study, individuals in the Paleolithic diet group had higher fat consumption and lower carbohydrate intake compared to those in the control diet group, and we found that decreased carbohydrate intake was associated with increased postprandial GIP levels. These results are in line with the findings of Sloth and colleagues, demonstrating lower postprandial GIP levels associated with 6 months of weight maintenance with a low-fat diet compared to a control diet with higher fat content (34). Therefore, the increased postprandial GIP levels in the Paleolithic diet group of our study could either be due to altered diet composition or to a more pronounced weight loss.

As discussed earlier, it is suggested that glucagon may also decrease the appetite – even more so when it acts together with GLP-1 (35, 36), but the influence of weight loss on glucagon secretion in healthy individuals without diabetes has not been extensively studied. Iepsen and colleagues found no changes in fasting or postprandial glucagon levels in healthy obese individuals during 8 weeks on a very low-calorie diet and 1 year of body weight maintenance (14). In our study, both diet groups exhibited increased postprandial glucagon, which was not associated with weight loss or macronutrient composition of the diet. Examination of the postprandial glucagon responses (Fig. 2I and J) revealed a tendency towards increased fasting glucagon levels after 6 and 24 months of intervention, moving the whole graph slightly upward. However, glucose-induced suppression was unaltered.

Glucagon maintains stable blood glucose levels during fasting by promoting hepatic glucose production, mostly through glycogenolysis. Both diets in our study induced weight loss via reduced calorie intake (despite *ad libitum* intake according to study design), which may in itself increase fasting glucagon secretion. Some participants probably had a very low-calorie intake the days before measurement causing a catabolic state during examination, as supported by increased levels of the ketone body β-hydroxybutyrate in concert with higher fasting non-esterified fatty acids. Therefore, the less suppressed postprandial glucagon levels during our intervention can be interpreted as a physiological reaction to a catabolic state. Indeed, we identified a significant association between increased ketone bodies and increased fasting glucagon levels. The altered protein and fat intake in both diet groups may influence both ketogenesis and amino acid metabolism.

The participants in this study were metabolically healthy: glucose and insulin, both fasting and postprandially, were within the normal range. This explains why glucose and insulin showed only minor changes after the intervention despite the significant weight loss. It is important to have this healthy study population in mind when interpreting the results of GLP-1, GIP and glucagon. Individuals with impaired glucose tolerance or diabetes would have had higher glucagon levels and lower GLP-1 levels at study start which would have influenced the weight loss effect on these hormones (10, 20).

The present study has several limitations. First, it is difficult to disentangle the effects of weight loss and diet composition, respectively, on hormonal changes. Notably, the Paleolithic diet causes a more pronounced weight loss compared to the control diet. The correlation analyses can therefore not infer causality. Second, not all enrolled women completed the study. Our analysis includes only the individuals who did not leave the study protocol. The women who remained in the study probably achieved greater weight loss than those who left the study. This fact introduces selection bias but gives us the possibility to study the effect of more pronounced weight loss. We cannot exclude the possibility that hormone levels differed in individuals who discontinued the study. Third, we examined incretin and glucagon levels using an oral glucose tolerance test, but a mixed meal test would have provided a more physiological examination. Fourth, our study protocol only included examination of incretin secretion. Incretin sensitivity may also be important for weight loss and weight maintenance, but was not assessed in this study. Fifth, incretin hormone and glucagon levels were analysed in batch after completion of the study. Because of the long study duration, storage time differed between baseline and 2-year samples which may influence hormone levels. Incretin and glucagon levels were analysed 4 years after study completion. Glucagon levels are mostly reduced during the first month of storage and much less during the following months (37). GLP-1 levels are more or less stable during 1 year of storage (37). Due to the long storage time after completion of the study, the study duration of 2 years is less important for the analysis results.

In summary, our study suggests that weight loss increases postprandial GLP-1 levels, which promote satiety and help to maintain body weight after weight loss. During time periods where metabolism is in a slightly catabolic state, less suppressed glucagon levels may enhance this effect of GLP-1.

## Declaration of interest

The authors declare that there is no conflict of interest that could be perceived as prejudicing the impartiality of this study.

## Funding

This work was supported by The Swedish Council for Working Life and Social Research (grant numbers 2006-0699, 2010-0398); the Swedish Research Council (grant number K2011-12237-15-16); the Swedish Heart and Lung Foundation; King Gustaf V and Queen Victoria’s Foundation; the Swedish Diabetes Research Foundation; the County Council of Västerbotten and Umeå University, Sweden.

## Author contribution statement

M R, C M, B L, C L and T O designed the study. J O, J J H and T O wrote the manuscript. All authors analysed and interpreted the data, revised the manuscript and gave their final approval for publication.
